# Retrospective Analysis of Structural Disease Progression in Retinitis Pigmentosa Utilizing Multimodal Imaging

**DOI:** 10.1038/s41598-017-10473-0

**Published:** 2017-09-04

**Authors:** Thiago Cabral, Jesse D. Sengillo, Jimmy K. Duong, Sally Justus, Katherine Boudreault, Kaspar Schuerch, Rubens Belfort, Vinit B. Mahajan, Janet R. Sparrow, Stephen H. Tsang

**Affiliations:** 1 Jonas Children’s Vision Care, and the Bernard & Shirlee Brown Glaucoma Laboratory, Department of Ophthalmology, Columbia University, New York, NY USA; 2 Edward S Harkness Eye Institute, New York-Presbyterian Hospital, New York, NY USA; 3 Department of Ophthalmology, Federal University of Espírito Santo, Vitória, Brazil; 4 Department of Ophthalmology, Federal University of São Paulo, São Paulo, Brazil; 5 State University of New York Downstate Medical Center, Brooklyn, NY USA; 6 Department of Biostatistics, Columbia University, New York, NY USA; 7 Department of Ophthalmology, University of Montreal, Montreal, QC Canada; 8 Omics Laboratory, Byers Eye Institute, Department of Ophthalmology, Stanford University, Palo Alto, CA USA; 9 Department of Pathology & Cell Biology, Institute of Human Nutrition, College of Physicians and Surgeons, Columbia University, New York, NY USA

## Abstract

In this report, we assess the natural progression rate of retinitis pigmentosa (RP) over an average of three years using spectral-domain optical coherence tomography (SD-OCT) and short wavelength fundus autofluorescence (SW-AF). Measurement of the ellipsoid zone (EZ) line width and hyperautofluorescent ring diameters was performed in 81 patients with RP in a retrospective, longitudinal fashion. Rate of structural disease progression, symmetry between eyes, and test-retest variability were quantified. We observed on average, EZ-line widths decreased by 140 µm (5.2%, p < 0.001) per year, and average horizontal and vertical hyperautofluorescent ring diameters decreased by 149 µm (3.6%, p < 0.001) and 120 µm (3.9%, p < 0.001) per year, respectively. The 95th percentile of this cohort had differences in progression slopes between eyes that were less than 154 µm, 118 µm, and 132 µm for EZ-line width and horizontal and vertical ring diameters, respectively. For all measures except horizontal ring diameter, progression rates were significantly slower at end-stage disease. From our data, we observed a statistically significant progression rate in EZ line width and SW-AF ring diameters over time, verifying the utility of these measurements for disease monitoring purposes. Additionally, calculated differences in progression slopes between eyes may prove useful for investigators evaluating the efficacy of unilateral treatments for RP in clinical trials.

## Introduction

Retinitis pigmentosa (RP) is an inherited retinal disorder that causes progressive photoreceptor death and subsequent irreversible vision loss. Affecting approximately 1 in 4000 individuals worldwide, RP patients typically present with night blindness followed by a constricting visual field and eventually, visual impairment or severe blindness^1, 2^. Mutations in more than sixty-seven genes have been identified to cause non-syndromic RP and^3–5^, despite this genetic heterogeneity, a similar end-result of photoreceptor cell dysfunction and cell death results^6^. Specialized genetic counseling and optimizing residual vision remain central to the management of RP, as there is currently no treatment that reverses disease progression^7^. However, numerous gene-, drug-, and cell-based therapy clinical trials are underway for inherited retinal degenerations including RP, highlighting the need for studies defining natural disease history. A previous study from our group by Sujirakul *et al*.^8^ observed ellipsoid zone (EZ)-line width and hyperautofluorescent ring diameters to be a reliable measure for RP progression. In a large cohort over a mean follow-up of two years, the investigators quantified disease progression rate and assessed for influence of other factors. Continued long-term tracking of RP disease progression yields important data that can serve as a metric for future clinical trials and patient counseling, and thus the present analysis is a follow-up to the previous study over a longer follow-up period.

Traditionally, tests of visual or retinal function were first employed to track disease progression, such as visual acuity, visual fields, and electroretinography^9^. Visual acuity decreases slowly with age in comparison to visual field, in which concentric visual field loss is more progressive and typically will spare a portion of the patient’s central vision. Mid-peripheral scotomas are also observed in many patients, likely because this area of the fundus contains the highest rod density^9, 10^. Although these tests correlate to the perceptual experience of the patient, intersessional visual acuity and field measurements are variable. Such measurements for RP patients require larger changes to confidently identify progression and benefit from a *single* experienced operator administering the test; this is likely due to the high subjectivity and low test-retest reliability of these metrics^11–13^. Furthermore, variability of visual acuity and field increases with disease severity in RP, which is pertinent to RP clinical trials, as most enrolled patients have advanced-stage disease^14^.

**Figure 1:**
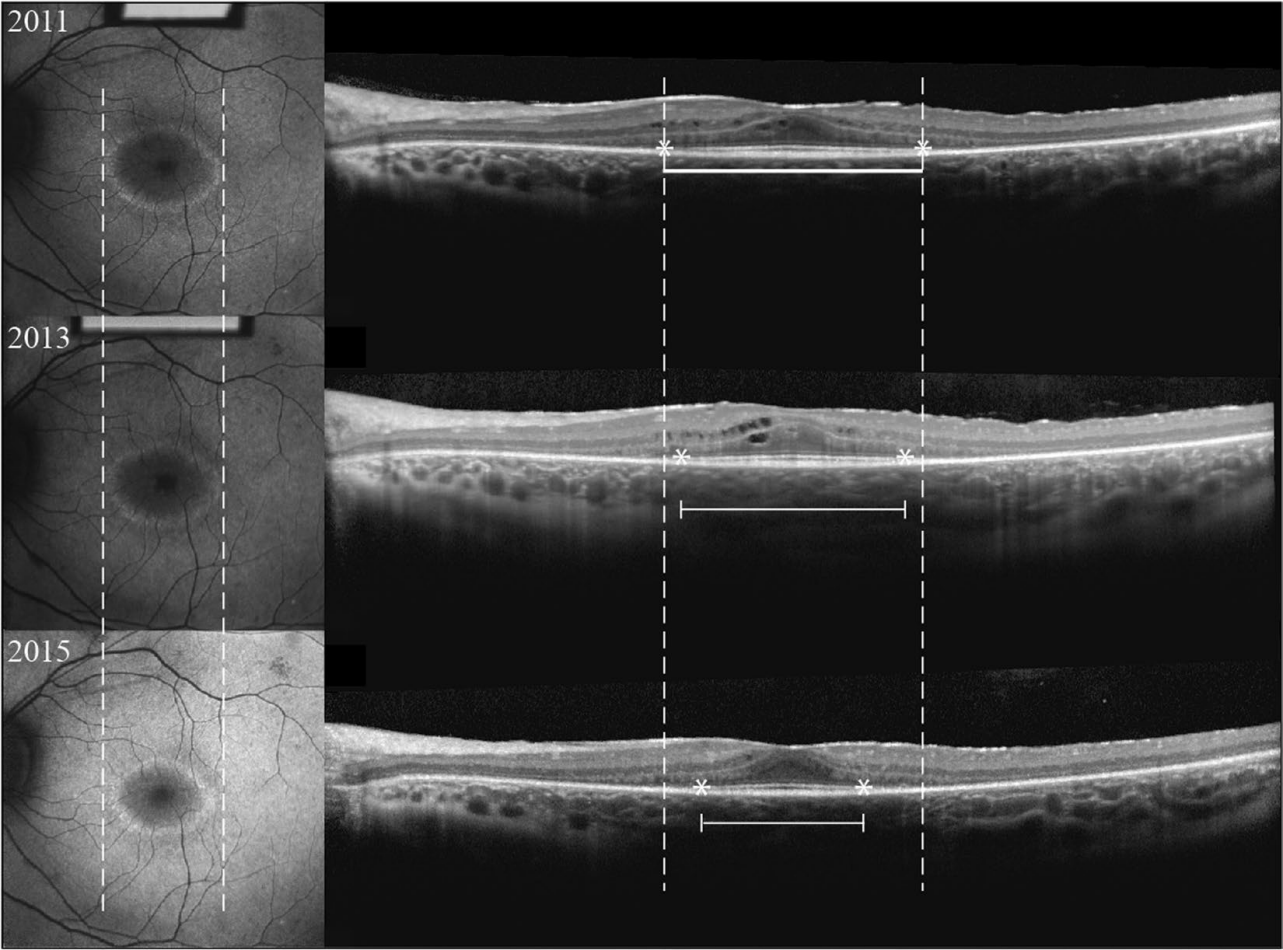
Longitudinal SD-OCT and SW-AF images of a 16-year-old man with autosomal dominant retinitis pigmentosa associated with a mutation in the *PRPF31* gene. SW-AF images (left column) show characteristic hyperautofluorescent rings and SD-OCT (right column) images visualize the EZ line. Solid lines; sample measurement. Dashed lines; initial measurement. Asterisk; endpoints on EZ-line.

Objective visual function is attained using the electroretinogram (ERG) and is more sensitive. Inherited retinal disease can manifest on ERG at an early-stage of disease prior to any structural change and provides a prognosis for future visual function^15^. Physicians alter the adaptation state and light stimulus to yield different information from the ERG. Although this modality has been useful in monitoring disease progression and treatment response^16^, its intrinsic variability between sessions and large threshold for significant change limit its use over short intervals^17–21^. As such, we decided to take advantage of two *non-invasive* imaging modalities. High resolution SD-OCT visualizes the ellipsoid zone of the retina, an area that approximates the perceptual experience of a patient, as it correlates to visual field boundaries and can be used to monitor progression^22–27^ (Fig. 1). Short wavelength fundus autofluorescence (SW-AF) imaging of the retina is another technique used to assess inherited retinal disease and takes advantage of a major fluorophore, lipofuscin, that increasingly accumulates in the retinal pigment epithelium in diseased states^28^. It was previously observed that some RP patients possess a progressively constricting hyperautofluorescent ring on SW-AF, which correlates with worsening of visual function over time as measured by pattern ERG^29^. Subsequent analyses have confirmed this relationship to visual function and structural changes, such as abnormalities of the EZ on SD-OCT^28, 30–42^. Here, we provide progression rates for RP over a three-year average follow-up utilizing SD-OCT and SW-AF imaging.

## Results

### Clinical data

Eighty-one patients were followed for an average of 3.1 years (SD 1.7 years; median of 3 years with an interquartile range of 1.8, 4.6), defined by the amount of time between the first and last visit in which the right eye was imaged with SD-OCT imaging, as this imaging modality was utilized more frequently compared to SW-AF. In our cohort, 44 (54%) patients were male and 37 (46%) were female. Of these patients, 24 (30%) were diagnosed with autosomal dominant RP (ADRP), 53 (65%) were diagnosed with autosomal recessive RP (ARRP), and 4 (5%) were diagnosed with X-linked RP (XLRP). Twelve patients (15%) had a history of bilateral hearing loss consistent with Usher Syndrome. The average age of patients at first visit was 39 ± 19 years old (mean ± SD). Clinical and genetic data is summarized in Table 1 and distribution of follow-up time is shown in Table 2. Regarding imaging of the EZ-line, 78 of the patients (96%) had at least one eye with an EZ within the SD-OCT field of view. The 3 patients with EZs that extended outside the SD-OCT field of view each had a measurable hyperautofluorescent ring. Different SW-AF patterns were found as expected. Eleven of the patients (13.6%) included in this study were observed to have cystoid macular edema (CME).

**Table 1:** Summary of clinical and genetic characteristics of the cohort.

Inheritance	Subjects	Proportion	Genes Implicated (# of Patients)
ARRP	41	50.6%	*USH2A* (2), *PDE6B* (2), *PDE6A* (2), *CNGB1* (2), *MERTK* (2), *MAK1* (1), *NPHP1* (1), *EYS* (1), *CRB1* (1), *RGR* (1), Unknown (26)
ADRP	24	29.6%	*RHO* (4)*, PRPF31* (3), *RP1* (3), Unknown (14)
Usher Syndrome	12	14.8%	*USH2A* (5), *GPR98* (1), *PCDH15* (1), Unknown (5)
XLRP	4	5%	*RPGR* (3), Unknown (1)

*ARRP* autosomal recessive retinitis pigmentosa, *ADRP* autosomal dominant retinitis pigmentosa, *XLRP* X-linked retinitis pigmentosa.

**Table 2:** Distribution of follow-up time as defined by ellipsoid zone measurements in the right eye.

n	Mean (yrs)	Standard Deviation	Quantiles				
			Minimum	25^th^	Median	75^th^	Maximum
77	3.1	1.7	0.00	1.8	3.0	4.6	6.1

### Rate of progression

**Figure 2:**
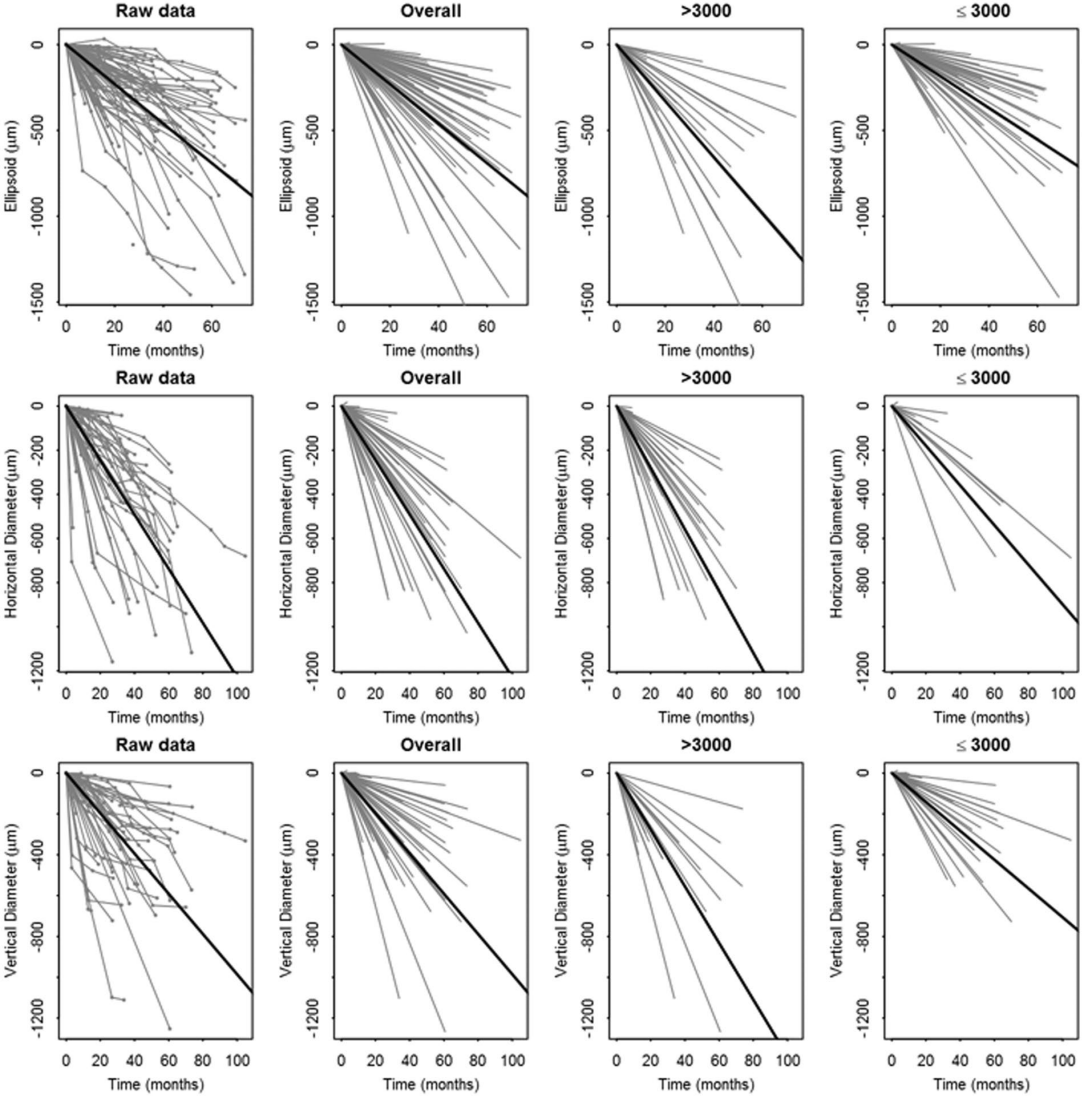
Trajectory of progression. Graphical representation of structural progression of each RP patient for EZ line width (first row), horizontal ring diameter (second row), and vertical diameter (third row). The raw data for each individual patient, followed time at each visit, is represented in the first column. Overall progression (second column), progression for patients with a baseline measurement ≤3000 µm (third column), and progression for patients with a baseline measurement >3000 µm (fourth column) is illustrated as a linear approximation for each patient. Single bold line in each graph represents average progression.

EZ-line width and hyperautofluorescent ring diameters are presented graphically as a function of time (Fig. 2). Linear mixed models with random intercepts and slopes were used to estimate progression rate in this study. Progression rate analysis for the right and left eyes are shown in Tables 3 and SI, respectively. The estimated average shortening of EZ-line width amongst right eyes was 140 ± 12 μm per year (mean ± SE, *p* < 0.001), representing approximately 0.5 degrees of visual field per year. Similarly, the estimated mean constriction of horizontal and vertical diameter was 149 ± 15 μm and 120 ± 14 μm per year (*p* < 0.001), respectively. Similar rates were observed in left eyes (Table SI). Progression rates were slower for patients with EZ-lines and ring diameters that were ≤ 3000 μm in size at baseline, with EZ-line width, horizontal diameter, and vertical diameter shortening at an average rate of 113 ± 14 μm, 109 ± 27 μm, 86 ± 14 μm per year in the right eye, respectively. For baseline measurements > 3000 μm, progression rates were faster, with EZ-line width, horizontal diameter, and vertical diameter shortening at an average rate of 200 ± 20 μm, 170 ± 17 μm, 169 ± 19 μm per year in the right eye, respectively (Table 3). Again, similar results were observed in left eyes (Table S1).

**Table 3:** Rate of progression in the right eye of advanced-stage retinitis pigmentosa calculated using spectral-domain optical coherence tomography and fundus autofluorescence.

Outcome	Progression ± SE (µm/year)	Progression (degrees/year)	*P* Value
Ellipsoid zone overall	140 ± 12	0.49	<0.001
Baseline ≤ 3000 µm	113 ± 14	0.40	<0.001
Baseline > 3000 µm	200 ± 20	0.70	<0.001
Horizontal diameter overall	149 ± 15	0.52	<0.001
Baseline ≤ 3000 µm	109 ± 27	0.38	<0.001
Baseline > 3000 µm	170 ± 17	0.60	<0.001
Vertical diameter overall	120 ± 14	0.42	<0.001
Baseline ≤ 3000 µm	86 ± 14	0.30	<0.001
Baseline > 3000 µm	169 ± 19	0.60	<0.001

*SE* standard error.

### Reliability of measurements

**Figure 3:**
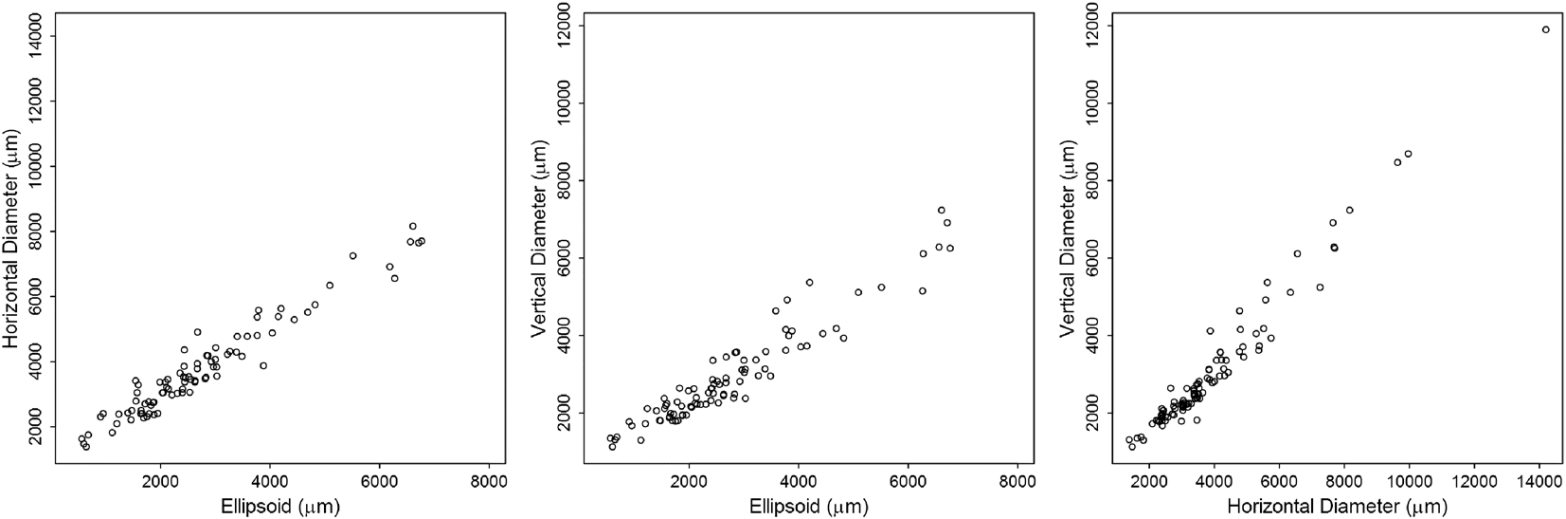
Correlation between measurements for initial visit. Each of the three structural measurements were plotted as a function of the other two measurements. Scatterplots show high correlation between EZ-line width and both SW-AF ring diameters (first and second panel), and between horizontal and vertical ring diameters (third panel).

To assess whether the three measurements were reliable, they were analyzed using descriptive statistics (Table 4) and intraclass correlation. Of note, the 95^th^ percentile of the difference between test and retest was less than 74 μm for EZ-line width, 77 μm for horizontal diameter, and 67 μm for vertical diameter. High intraclass correlation of each measurement was observed, indicating high reliability, specifically 0.9998 for EZ-line width, 0.9998 for horizontal diameter, and 0.9998 for vertical diameter. The three measurements are strongly correlated to each other during the first patient visit (Fig. 3): r = 0.97 between EZ-line width and horizontal diameter, r = 0.95 for EZ-line width and vertical diameter, and r = 0.98 for horizontal diameter and vertical diameter.

**Table 4:** Descriptive statistics of the absolute difference between test and retest measurements for structural imaging parameters used to monitor retinitis pigmentosa progression. All units are µm.

	Number of images	Mean ± SD	Lower Quartile	Median	Upper Quartile	95^th^ Percentile
Ellipsoid zone width	539	17 ± 28	4	7	17	74
Horizontal diameter	336	20 ± 37	3	8	19	77
Vertical diameter	340	18 ± 27	4	8	20	67

*SD* standard deviation.

### Symmetry of disease progression between eyes

Descriptive statistics was used to compare progression rates between eyes of the patient (Table 5). The absolute difference between slopes of progression in each eye of every patient was calculated. Of note, the 95^th^ percentile of the differences in slope of progression between each eye were 154 μm, 118 μm, and 132 μm for EZ-line width, horizontal diameter, and vertical diameter respectively.

**Table 5:** Descriptive statistics of the absolute difference between slopes of rate of progression between eyes measured with structural imaging parameters in retinitis pigmentosa. All units are µm.

	Number of patients	Mean ± SD	Lower Quartile	Median	Upper Quartile	95^th^ Percentile
Ellipsoid zone width	73	54 ± 47	19	39	78	154
Horizontal diameter	47	50 ± 48	11	36	74	118
Vertical diameter	47	37 ± 47	8	21	41	132

*SD* standard deviation.

### Examining for possible interactions

The effects of age, gender, mode of inheritance, and initial baseline measurement on progression rate were all evaluated by fitting linear mixed models of disease progression with time, these factors, and interactions of these factors with time (Tables SII–SV). Examining the estimate for the interaction term allows us to investigate whether progression varies by another factor. Age, gender, and mode of inheritance did not significantly affect progression rate in our patient cohort, similar to our previous investigation (Tables SII, SIII, SIV, respectively). Disease progression rate was observed to be a function of initial baseline measurement. We examined this in two ways, dichotomizing baseline values at 3000 µm (Table 3 and SV) and keeping baseline as a continuous value (Table SVI). For patients with baseline measurements >3000 μm, the average progression rate of the right and left eye was calculated to be significantly faster for ellipsoid zone line width and vertical ring diameter. Similar results were observed for the left eye (Tables SI and SV). For horizontal ring diameter, the p-value for the interaction terms were 0.06 and 0.32 for right eye and left eye respectively. (Table 3 and SV). When using continuous baseline values, results for ellipsoid zone line width and vertical ring diameter were similar, i.e., the interactions were also significant. For horizontal ring diameter, the p-values for the interaction terms were 0.002 and 0.13 for right eye and left eye respectively.

## Discussion

Overall, this study observed that EZ-line width measured by SD-OCT and hyperautofluorescent ring diameters measured with fundus SW-AF can detect progression in RP patients over a mean follow-up of 3 years, with each measurement highly correlated to the other; this parallels results of a two-year follow-up study performed by our group^8^. Progression rates were assessed using linear mixed models and comparable between the two studies. Although RP is thought to exhibit an exponential decay in degeneration, linear modeling is appropriate for approximating short intervals such as three years^8^. The mean annual decrease from average baseline EZ-line width was 5.2% per year, and 3.6% and 3.9% per year for horizontal and vertical hyperautofluorescent ring diameter; this is consistent with our previous report of 4.9%, 4.1%, and 4.0% per year, respectively. Although different statistical methods have been employed in a variety of published studies monitoring annual changes in functional and structural loss in RP patients, our results approximate previous findings^24, 25, 37, 44^. Given these data, EZ-line width and hyperautofluorescent ring diameters may serve as longitudinal outcome measures in clinical trials. Our study provides an estimated annual disease progression, which is useful for patient counseling and assessing efficacy of new treatments.

Despite that functional tests may appear subsidiary for tracking disease progression compared to objective structural measurements, they remain important. Representing a patient’s functional experience through subjective testing such as best-corrected visual acuity and visual fields provides useful information to clinicians. Large changes in visual function determined under consistent testing environments may prove significant as an outcome measure in clinical trials^11, 12^. ERGs remain useful for providing an objective visual function prognosis in patients with RP. For example, it is estimated that less than 0.5 µV seen on a 30 Hz-flicker ERG can be considered ‘virtual blindness’^43^. Voltages measured on 30 Hz-flicker can be used to estimate years left of useful vision, information which is invaluable for patient counseling^43^.

In gene and cell-based therapy clinical trials, one eye typically serves as a control while the contralateral eye receives treatment, usually in the form of a subretinal injection. When assuming disease progression is symmetric between eyes, this provides investigators the unique opportunity to compare the treated eye to a near-ideal control. However, previous studies have estimated that 10–14.3% of patients have asymmetry when quantified with SD-OCT or SW-AF^43, 45^. Our previous two-year study estimated that 20% of patients have asymmetric progression^8^. Each study uses different statistical methods for measuring and defining asymmetry, which makes comparing cohorts difficult. We believe a more useful way of comparing both eyes may be with descriptive statistics. For this cohort, we observed that 95% of this cohort had less than a 154 µm per year difference in progression rate for EZ-line width. Horizontal and vertical ring diameters were less than a difference of 118 µm and 132 µm per year between eyes. Instead of defining asymmetry, investigators can use this data to cautiously assess the results of clinical trials and determine how large of a difference between the treated and control eye is necessary before considering a therapy to be efficacious in delaying structural progression.

Our study used mixed effect models to assess the effect of other variables on disease progression. For our cohort, we did not observe an effect of age or gender alone on disease progression rate, as anticipated. Progression rates did significantly differ when assessed as a function of initial structural measurement. For all structural measurements utilized for both eyes, except horizontal ring diameters, progression rates were significantly faster when initial baseline measurement was > 3000 μm. This is consistent with the fact that the natural history of RP progresses in an exponential decay^43^. Of note, progression did not vary significantly as a function of mode of inheritance. It is previously reported that XLRP has a faster rate of disease progression^44^. In our study, XLRP did not have a significantly faster disease progression rate. However, the small number (n = 4) for XLRP diminishes the confidence of this interpretation.

Some limitations to using SD-OCT and SW-AF as outcome measures include the presence of cystoid macular edema (CME), which blurs the EZ line, and the fact that patients do not always have an easily quantifiable hyperautofluorescent ring on SW-AF^8^. Of note, 13.6% of patients at initial visit in our cohort exhibited CME. No patient had a clinically significant staphyloma, although curvature of the posterior pole was not accounted for with the measurement utilized. This may cause an underestimation of changes in EZ-line width, as curvature of the posterior pole will distort measurements of the vertical component of changes in EZ line length. For patients in which the disease has not reached the last 30 degrees of the posterior pole, newer OCT techniques such as swept source will need to be employed as opposed to SD-OCT. In contrast, SW-AF imaging can image a wider field. This highlights the importance of a multi-modal assessment of RP patients. Other challenges include the genetic heterogeneity of RP, which is well-documented^1, 6, 46, 47^, and may make the interpretation of natural progression studies difficult. In our cohort, 35 patients (43%) harbored variants consistent with disease phenotype and family history, which is 8% more than the previous study. Certain mutations may manifest with a progression that is not similar to the average projection of large cohorts like ours, which encompass RP patients with differing molecular diagnoses. Additionally, many recessive RP cases are compound heterozygous, so patients may have a unique progression rate depending on the severity of each mutation. Only 6 patients (7%) in our study have yet to undergo either ffERG or genetic testing. However, each of these patients experienced symptoms and clinical progression strongly consistent with a rod-cone dystrophy. The authors recognize that some conditions may masquerade as RP prior to ffERG or genetic testing. Due to the genetic heterogeneity of RP, sub-group analyses of large cohorts or multi-center natural progression studies for a specific gene-associated disease are needed but challenging for rare diseases, and they may be more useful for gene therapy-based trials in which enrolled patients share a gene-specific RP diagnosis. Further studies with longer follow-up and correlation to functional measures of vision loss will continue to provide important analyses of disease progression in preparation for future clinical trials.

## Methods

### Subjects

The current investigation is a follow-up to Sujirakul *et al*.^8^ and contains overlapping patients. However, all raw data and analyses were newly acquired with an increased number of subjects, genotypes, images and longer follow-up period. All subjects provided informed consent to participate in this study, which was approved by the Columbia University Internal Review Board and adhered to the tenets of the Declaration of Helsinki. None of the data presented in this study, including images and genetic testing results, is identifiable to individual patients. RP patients followed at the Harkness Eye Institute electrodiagnostics clinic were considered for inclusion, of which 81 met our criteria: (1) the patient must be monitored for at least two visits, (2) the patient must have bilateral RP. Patients were excluded if image quality was poor or if the EZ line was not visible in conjunction with an absence of a hyperautofluorescent ring. X-linked RP (XLRP) manifesting in females and paravenous RP were excluded from our study. All cases of RP were diagnosed clinically by an inherited retinal disease specialist (S.H.T.) based on presenting symptoms, family history, and fundus exam and subsequently supported by imaging or ffERG. Sixty-eight patients (84%) underwent ffERG testing, which showed tracings consistent with a rod-cone dystrophy. Of the 13 patients who did not undergo electroretinography, seven harbored variants in genes that were consistent with their phenotype and family history.

### Genetic analysis

Genetic testing was performed as described in our previous study^8^. Briefly, blood samples were drawn and sent to Oregon Health Sciences University. Extracted DNA was tested for published genes that cause RP by parallel sequencing on Illumina HiSeq platform with 100 bp paired-end reads. Dideoxy chain-terminating sequencing was subsequently used to confirm the mutation.

### Fundus autofluorescence and spectral-domain optical coherence tomography

All imaging was performed as described in our previous study^8^. Briefly, SW-AF images were acquired at each visit on dilated patients using the Spectralis HRA + OCT device (Heidelberg Engineering, Heidelberg, Germany) at a resolution of 1536 × 1536 pixels and a field of view of 30 degrees. For large hyperfluorescent rings, a 55-degree field of view was acquired. A 521 nm barrier filter was used to filter emitted light after excitation with a wavelength of 486 nm. The external boundary of the ring, which is more distinct than the internal boundary, was measured manually using the inbuilt software (Heidelberg Eye Explorer, software version 1.9.10.0, Heidelberg, Germany). One axis, vertical or horizontal, was measured if the patient had a hyperautofluorescent arc or a ring that fell outside of the optic nerve, making one diameter unmeasurable. At each visit, SD-OCT images were acquired using the Spectralis HRA + OCT device (Heidelberg Engineering, Heidelberg, Germany) with an 870 nm light source and automatic real-time registration program of both an SD-OCT and infrared reflectance (IR-R) image. Horizontal scans taken through the fovea provided visualization of the EZ-line and was measured manually using spectralis software. All SD-OCT images were acquired in high resolution mode (9 mm scans, ART, average of a minimum of 50 images).

### Statistical analyses

Measurements were obtained from SD-OCT and fundus SW-AF images recorded for all visits of each patient in the cohort. EZ-line width and horizontal and vertical hyperfluorescent ring diameters were measured twice by the first author (T.C.) one week apart (Fig. 1). Progression rates and test-retest reliability of measurements were calculated as previously described^8^. Reliability was assessed with descriptive statistics for differences between test-retest values and by calculating intraclass correlation coefficients of test-retest measurements. Pearson correlations were used to compare structural measurements for the initial visit. Progression rates were estimated using linear mixed models with random intercepts and slopes, where the random slope for a subject was his/her estimated change over time. Left and right eyes were compared by calculating the absolute difference between the random slopes and using descriptive statistics to quantify the asymmetry within various percentiles. Linear mixed models were fit for other factors of interest such as: age, gender, mode of inheritance, and initial baseline measurement. Initial baseline measurement was assessed as a dichotomized (Table SV) and continuous (Table SVI) variable. The models which were fit included time, the factors of interest, and the interaction of this factor with time. Statistical analyses were done using R version 3.3.1, and also utilizing utilized *nlme* and *lme4* sub packages (Vienna, Austria).

## Electronic supplementary material

Supplemental Tables
