# Supplementary material for: Retrospective Analysis of Structural Disease Progression in Retinitis Pigmentosa Utilizing Multimodal Imaging
Source: Sci Rep. 2017 Sep 4;7:10347. doi: 10.1038/s41598-017-10473-0 (PMC5583352; doi:10.1038/s41598-017-10473-0)
Supplement: Supplementary file 1 — Supplemental Tables [file 41598_2017_10473_MOESM1_ESM.doc]

**Retrospective Analysis of Structural Disease Progression in Retinitis Pigmentosa Utilizing Multimodal Imaging**

Thiago Cabral^1,2,3,4^, Jesse D Sengillo^1,2,5^, Jimmy K Duong^6^, Sally Justus^1,2^, Katherine Boudreault^1,2,7^, Kaspar Schuerch^2^, Rubens Belfort Jr^4^, Vinit B Mahajan^8^, Janet R Sparrow^2,9^, Stephen H Tsang^1,2,9*^

^1^Jonas Children’s Vision Care, and the Bernard & Shirlee Brown Glaucoma Laboratory, Department of Ophthalmology, Columbia University, New York, NY, USA

^2^Edward S Harkness Eye Institute, New York-Presbyterian Hospital, New York, NY, USA

^3^Department of Ophthalmology, Federal University of Espírito Santo, Vitória, Brazil

^4^Department of Ophthalmology, Federal University of São Paulo, São Paulo, Brazil

^5^State University of New York Downstate Medical Center, Brooklyn, NY, USA

^6^Department of Biostatistics, Columbia University, New York, NY, USA

^7^Department of Ophthalmology, University of Montreal, Montreal, QC, Canada

^8^Omics Laboratory, Byers Eye Institute, Department of Ophthalmology, Stanford University, Palo Alto, CA, USA

^9^Department of Pathology & Cell Biology, Institute of Human Nutrition, College of Physicians and Surgeons, Columbia University, New York, NY, USA.

*Corresponding author

Address Correspondence:

Stephen H Tsang, MD, PhD

Edward S. Harkness Eye Institute

635 West 165th Street, 5th floor, Box 212, New York, NY 10032

Phone: (212) 342-1189 / Fax: 212-305- 4987 / Email: Sht2@columbia.edu

**SUPPLEMENTAL TABLES**

*Supplementary Table SI.* Rate of progression in the left eye of advanced-stage retinitis pigmentosa calculated using spectral-domain optical coherence tomography and fundus autofluorescence.

| Outcome | Progression ± SE (µm/year) | Progression (degrees/year) | *P* Value |
| --- | --- | --- | --- |
| Ellipsoid zone overall | 137 ± 14 | 0.48 | <0.001 |
| Baseline ≤ 3000µm | 94 ± 14 | 0.33 | <0.001 |
| Baseline > 3000µm | 223 ± 22 | 0.79 | <0.001 |
| Horizontal diameter overall | 129 ± 14 | 0.45 | <0.001 |
| Baseline ≤ 3000µm | 109 ± 25 | 0.38 | <0.001 |
| Baseline > 3000µm | 139 ± 16 | 0.49 | <0.001 |
| Vertical diameter overall | 111 ± 13 | 0.39 | <0.001 |
| Baseline ≤ 3000µm | 79 ± 16 | 0.28 | <0.001 |
| Baseline > 3000µm | 174 ± 20 | 0.61 | <0.001 |

*SE* standard error

*Supplementary Table SII.* Analysis of the effect of age on progression rate for patients with retinitis pigmentosa monitored with structural measurements.

| Outcome | Regression coefficient (SE) | P value |
| --- | --- | --- |
| Ellipsoid zone (OD) |  |  |
| Intercept | 2802 (483) | <0.001 |
| Age (y) | -3 (11) | 0.8 |
| Time (y) | -122 (28) | <0.001 |
| Age * time | -0.5 (0.6) | 0.5 |
| Ellipsoid zone (OS) |  |  |
| Intercept | 2365 (448) | <0.001 |
| Age (y) | 5.5 (10) | 0.6 |
| Time (y) | -126 (32) | <0.001 |
| Age * time | -0.3 (0.7) | 0.7 |
| Horizontal Diameter (OD) |  |  |
| Intercept | 4700 (713) | <0.001 |
| Age (y) | -17 (19) | 0.4 |
| Time (y) | -164 (34) | <0.001 |
| Age * time | 0.4 (0.9) | 0.6 |
| Horizontal Diameter (OS) |  |  |
| Intercept | 4598 (704) | <0.001 |
| Age (y) | -20 (19) | 0.3 |
| Time (y) | -144 (31) | <0.001 |
| Age * time | 0.4 (0.8) | 0.6 |
| Vertical Diameter (OD) |  |  |
| Intercept | 3256 (517) | <0.001 |
| Age (y) | -4 (14) | 0.8 |
| Time (y) | -142 (32) | <0.001 |
| Age * time | 0.6 (0.8) | 0.4 |
| Vertical Diameter (OS) |  |  |
| Intercept | 3570 (627) | <0.001 |
| Age (y) | -15 (17) | 0.4 |
| Time (y) | -123 (30) | <0.001 |
| Age * time | 0.3 (0.8) | 0.7 |

*Supplementary Table SIII*. Analysis of the effect of gender on progression rate for patients with retinitis pigmentosa monitored with structural measurements.

| Outcome | Regression coefficient (SE) | P value |
| --- | --- | --- |
| Ellipsoid zone (OD) |  |  |
| Intercept | 2781 (278) | <0.001 |
| Female | -193 (419) | 0.6 |
| Time (y) | -129 (16) | <0.001 |
| female * time | -27 (25) | 0.3 |
| Ellipsoid zone (OS) |  |  |
| Intercept | 2549 (260) | <0.001 |
| Female | 65 (384) | 0.9 |
| Time (y) | -116 (18) | <0.001 |
| female * time | -46 (27) | 0.1 |
| Horizontal Diameter (OD) |  |  |
| Intercept | 3928 (421) | <0.001 |
| Female | 442 (619) | 0.5 |
| Time (y) | -133 (20) | <0.001 |
| female * time | -36 (30) | 0.2 |
| Horizontal Diameter (OS) |  |  |
| Intercept | 3733 (426) | <0.001 |
| Female | 400 (628) | 0.5 |
| Time (y) | -122 (18) | <0.001 |
| female * time | -16 (28) | 0.6 |
| Vertical Diameter (OD) |  |  |
| Intercept | 3073 (301) | <0.001 |
| Female | 74 (442) | 0.9 |
| Time (y) | -100 (18) | <0.001 |
| female * time | -43 (27) | 0.1 |
| Vertical Diameter (OS) |  |  |
| Intercept | 2915 (377) | <0.001 |
| Female | 360 (557) | 0.5 |
| Time (y) | -110 (18) | <0.001 |
| female * time | -3 (28) | 0.9 |

*Supplementary Table SIV*. Analysis of the effect of mode of inheritance on progression rate for patients with retinitis pigmentosa monitored with structural measurements.

| Outcome | Regression coefficient (SE) | P value |
| --- | --- | --- |
| Ellipsoid zone (OD) |  |  |
| Intercept | 2885 (374) | <0.001 |
| ARRP | -235 (457) | 0.6 |
| XLRP | -771 (990) | 0.4 |
| Time (y) | -146 (21) | <0.001 |
| ARRP * time | 9 (26) | 0.7 |
| XLRP * time | -1 (66) | 0.98 |
| Ellipsoid zone (OS) |  | |
| Intercept | 2901 (342) | <0.001 |
| ARRP | -424 (417) | 0.3 |
| XLRP | -980 (888) | 0.3 |
| Time (y) | -152 (24) | <0.001 |
| ARRP * time | 17 (30) | 0.6 |
| XLRP * time | 86 (72) | 0.2 |
| Horizontal diameter (OD) |  | |
| Intercept | 4189 (527) | <0.001 |
| ARRP | 51.3 (667) | 0.9 |
| XLRP | -1113 (1237) | 0.4 |
| Time (y) | -152 (24) | <0.001 |
| ARRP * time | 6 (32) | 0.9 |
| XLRP * time | -24 (67) | 0.7 |
| Horizontal diameter (OS) |  | |
| Intercept | 3866 (559) | <0.001 |
| ARRP | 174 (692) | 0.8 |
| XLRP | -676 (1249) | 0.6 |
| Time (y) | -130 (24) | <0.001 |
| ARRP * time | 4 (30) | 0.9 |
| XLRP * time | -21 (59) | 0.7 |
| Vertical diameter (OD) |  |  |
| Intercept | 3348 (363) | <0.001 |
| ARRP | -287 (467) | 0.5 |
| XLRP | -1031 (870) | 0.2 |
| Time (y) | -122 (22) | <0.001 |
| ARRP * time | 0.7 (29) | 0.98 |
| XLRP * time | 3 (62) | 0.96 |
| Vertical diameter (OS) |  | |
| Intercept | 3043 (495) | <0.001 |
| ARRP | 157 (612) | 0.8 |
| XLRP | -726 (1105) | 0.5 |
| Time (y) | -96 (24) | <0.001 |
| ARRP * time | -20 (30) | 0.5 |
| XLRP * time | -61 (58) | 0.3 |

*Supplementary Table SV.* Analysis the effect of a particular measurement’s size at baseline (dichomotized variable) on progression rate for patients with retinitis pigmentosa monitored with structural measurements.

| Outcome | Regression coefficient (SE) | P value |
| --- | --- | --- |
| Ellipsoid zone (OD) |  |  |
| Intercept | 1676 (135) | <0.001 |
| >3000µm | 2992 (241) | <0.001 |
| Time (y) | -113 (14) | <0.001 |
| >3000µm * time | -87 (25) | <0.001 |
| Ellipsoid zone (OS) |  |  |
| Intercept | 1684 (134) | <0.001 |
| >3000µm | 2877 (239) | <0.001 |
| Time (y) | -94 (14) | <0.001 |
| >3000µm * time | -129 (26) | <0.001 |
| Horizontal Diameter (OD) |  |  |
| Intercept | 2358 (517) | <0.001 |
| >3000µm | 2437 (609) | <0.001 |
| Time (y) | -109 (27) | <0.001 |
| >3000µm * time | -61 (32) | 0.06 |
| Horizontal Diameter (OS) |  |  |
| Intercept | 2347 (482) | <0.001 |
| >3000µm | 2426 (591) | <0.001 |
| Time (y) | -109 (25) | <0.001 |
| >3000µm * time | -30 (30) | 0.3 |
| Vertical Diameter (OD) |  |  |
| Intercept | 2189 (167) | <0.001 |
| >3000µm | 2400 (278) | <0.001 |
| Time (y) | -86 (14) | <0.01 |
| >3000µm * time | -83 (23) | <0.001 |
| Vertical Diameter (OS) |  |  |
| Intercept | 2148 (253) | <0.001 |
| >3000µm | 2904 (438) | <0.001 |
| Time (y) | -79 (16) | <0.001 |
| >3000µm * time | -95 (26) | <0.001 |

*Supplementary Table SVI.* Analysis the effect of a particular measurement’s size at baseline (continuous variable) on progression rate for patients with retinitis pigmentosa monitored with structural measurements

| Outcome | Regression coefficient (SE) | P value |
| --- | --- | --- |
| Ellipsoid zone (OD) |  |  |
| Intercept | 3 (15) | 0.9 |
| Baseline (µm) | 1 (0.004) | < 0.001 |
| Time (y) | -75 (21) | < 0.001 |
| Baseline * time | -0.03 (0.007) | < 0.001 |
| Ellipsoid zone (OS) |  |  |
| Intercept | -13 (14) | 0.4 |
| Baseline (µm) | 1 (0.005) | < 0.001 |
| Time (y) | -23 (23) | 0.3 |
| Baseline * time | -0.04 (0.007) | < 0.001 |
| Horizontal Diameter (OD) |  |  |
| Intercept | -10 (26) | 0.7 |
| Baseline (µm) | 1 (0.006) | < 0.001 |
| Time (y) | -66 (33) | 0.05 |
| Basline * time | -0.02 (0.007) | 0.002 |
| Horizontal Diameter (OS) |  |  |
| Intercept | -18 (19) | 0.3 |
| Baseline (µm) | 1 (0.004) | < 0.001 |
| Time (y) | -90 (30) | 0.003 |
| Baseline * time | -0.01 (0.007) | 0.13 |
| Vertical Diameter (OD) |  |  |
| Intercept | 15 (21) | 0.5 |
| Baseline (µm) | 1 (0.006) | < 0.001 |
| Time (y) | -34 (28) | 0.22 |
| Baseline * time | -0.03 (0.008) | < 0.001 |
| Vertical Diameter (OS) |  |  |
| Intercept | -19 (13) | 0.1 |
| Baseline (µm) | 1 (0.004) | < 0.001 |
| Time (y) | -51 (28) | 0.07 |
| Baseline * time | -0.02 (0.007) | 0.005 |
